# The acyltransferase Gpc1 is both a target and an effector of the unfolded protein response in *Saccharomyces cerevisiae*

**DOI:** 10.1016/j.jbc.2023.104884

**Published:** 2023-06-02

**Authors:** Victoria Lee Hrach, William R. King, Laura D. Nelson, Shane Conklin, John A. Pollock, Jana Patton-Vogt

**Affiliations:** Department of Biological Sciences, Duquesne University, Pittsburgh, Pennsylvania, USA

**Keywords:** acyltransferase, glycerophosphocholine, Gpc1, Ire1, lipid remodeling, lysophosphatidylcholine, membrane bilayer stress, phosphatidylcholine, phospholipid metabolism, unfolded protein response, yeast

## Abstract

The unfolded protein response (UPR) is sensitive to proteotoxic and membrane bilayer stress, both of which are sensed by the ER protein Ire1. When activated, Ire1 splices *HAC1* mRNA, producing a transcription factor that targets genes involved in proteostasis and lipid metabolism, among others. The major membrane lipid phosphatidylcholine (PC) is subject to phospholipase-mediated deacylation, producing glycerophosphocholine (GPC), followed by reacylation of GPC through the PC deacylation/reacylation pathway (PC-DRP). The reacylation events occur *via* a two-step process catalyzed first by the GPC acyltransferase Gpc1, followed by acylation of the lyso-PC molecule by Ale1. However, whether Gpc1 is critical for ER bilayer homeostasis is unclear. Using an improved method for C^14^-choline-GPC radiolabeling, we first show that loss of Gpc1 results in abrogation of PC synthesis through PC-DRP and that Gpc1 colocalizes with the ER. We then probe the role of Gpc1 as both a target and an effector of the UPR. Exposure to the UPR-inducing compounds tunicamycin, DTT, and canavanine results in a Hac1-dependent increase in *GPC1* message. Further, cells lacking Gpc1 exhibit increased sensitivity to those proteotoxic stressors. Inositol limitation, known to induce the UPR *via* bilayer stress, also induces *GPC1* expression. Finally, we show that loss of *GPC1* induces the UPR. A *gpc1Δ* mutant displays upregulation of the UPR in strains expressing a mutant form of Ire1 that is unresponsive to unfolded proteins, indicating that bilayer stress is responsible for the observed upregulation. Collectively, our data indicate an important role for Gpc1 in yeast ER bilayer homeostasis.

The endoplasmic reticulum (ER) is the site of secretory and membrane protein synthesis and folding as well as the location at which most membrane lipids are synthesized. Cells experiencing ER stress invoke the unfolded protein response (UPR), a conserved transcriptional program originally described as being activated by unfolded proteins accumulating in the ER lumen. More recent studies have made clear that ER bilayer stress (LBS) can also induce UPR (1, 2, 3, 4, 5, 6, 7, 8). Unlike the mammalian UPR which includes three signaling pathways (IRE1, PERK, and ATF6), the yeast UPR has a single pathway of induction through Ire1 (9). The Ire1 transmembrane protein, termed inositol-requiring enzyme, acts as a sensor for ER stress and is required for UPR activation in yeast. An amphipathic helix adjacent to the single transmembrane helix of Ire1 has recently been proposed to be crucial for detecting and responding to ER bilayer stress (1, 5), while the N-terminal luminal domain (LD) has been shown to detect misfolded and unfolded proteins (4). Following either mechanism of activation, Ire1 dimerizes, gaining endonuclease activity that splices *HAC1* mRNA (2), to produce a mature Hac1 transcription factor that binds to unfolded protein response elements (UPREs) in the promoter of UPR target genes. The UPR targets hundreds of genes, including those involved in protein degradation, protein folding, secretion, lipid metabolism, and others (4). Interestingly, the UPR transcriptional response diverges somewhat based on whether induction occurs *via* bilayer stress or proteotoxic stress (5), with some stressors activating both pathways (4). Ire1 constructs with modifications to their LDs have been used as tools for determining if perturbations that activate the UPR do so *via* bilayer stress, unfolded protein stress, or both (5, 8, 10). Perturbations shown to induce the UPR *via* bilayer stress include altered lipid saturation (11, 12), altered sterol content (11), inositol limitation (3, 5, 13), increased phophatidylmonomethylethanolamine content (14), and altered phosphatidylethanolamine/phosphatidylcholine (PE/PC) ratio (15, 16).

The maintenance of membrane bilayer homeostasis requires a complex set of metabolic reactions. The major phospholipid PC is synthesized *via* two major routes in *Saccharomyces cerevisiae* (17). In the absence of exogenous choline, PC synthesis occurs primarily through the sequential methylation of PE (Fig. 1). When choline is supplied exogenously or released through catabolism, PC can be synthesized *via* the CDP-choline (Kennedy) pathway (18). We have recently uncovered a third pathway for PC synthesis, the PC deacylation/reacylation pathway (PC-DRP). In PC-DRP (Fig. 1), PC undergoes complete deacylation by type B phospholipases, primarily Nte1 and Plb1 (19, 20, 21) to generate free fatty acids and glycerophosphocholine (GPC). The GPC acyltransferase Gpc1 then acylates GPC at the *sn*-1 position to form lyso-PC, followed by a second acylation by Ale1 to generate a new PC molecule (22, 23). Ale1 is not limited to PC-DRP but is a broad-specificity lyso-PC acyltransferase (24, 25, 26).

**Figure 1:**
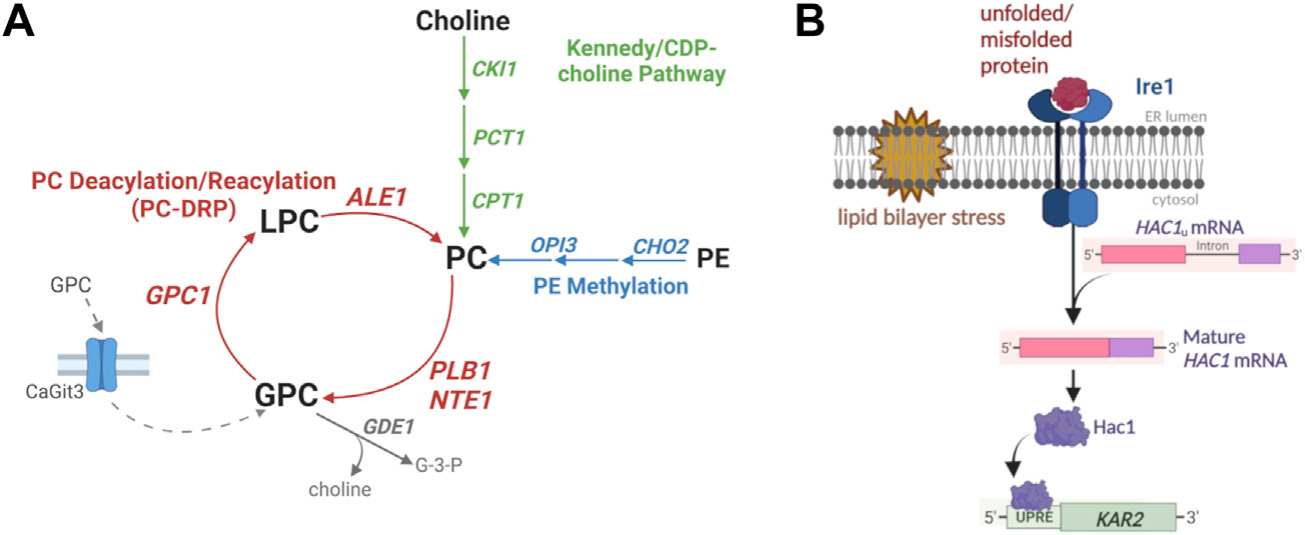
**PC-DRP and UPR pathways.***A*, simplified model of PC metabolism in *S. cerevisiae*. The *dashed line* indicates uptake of GPC *via* heterologous expression of CaGit3 transporter for labeling experiments. Genes encoding structural enzymes are in italics. *B*, the UPR is responsive to both unfolded proteins and bilayer stress, both of which result in *HAC1* splicing. *KAR2* is a chaperone and target of the UPR. Figure created in Biorender.com. CaGit3, *Candida albicans* Git3 transporter; GPC, glycerophosphocholine; LPC, lysophosphatidylcholine; PC, phosphatidylcholine; PC-DRP, PC Deacylation/Reacylation Pathway.

In *S. cerevisiae*, C16 and C18 fatty acids predominate, with each acyl chain typically having one or zero unsaturations (27). Thus, the four major PC species in yeast and their respective acyl chain combinations are 32:1PC (C16:0, C16:1); 32:2PC (C16:1, C16:1); 34:1PC (mostly C16:0 and C18:1); and 34:2PC (C16:1, C18:1) (28, 29, 30). PC species profile can vary depending upon biosynthetic pathway and media conditions (31). We have shown previously that a mutant strain lacking Gpc1, which catalyzes the committed step in GPC reacylation, displays a decrease (roughly 20%) in monounsaturated PC species (32:1PC and 34:1PC) and a concomitant increase in diunsaturated PC species (32:2PC and 34:2PC) when grown under a condition that upregulated *GPC1* expression (inositol-free media) (23). Thus, Gpc1, which catalyzes the committed step in the reacylation sequence and was discovered in our laboratory, provides the cell with both a two-step PC resynthesis route and the potential for acyl chain remodeling (22, 23).

Although the UPR upregulates the expression of several lipid-related genes, loss-of-function mutations in those genes do not necessarily result in UPR induction (32). Focusing on PC metabolism, *CHO2, OPI3, CKI1*, and *CPT1* (see Fig. 1) are UPR targets (4, 5, 32, 33, 34, 35, 36). However, only the loss of *OPI3* or *CHO2* results in LBS and UPR induction. *OPI3*, which catalyzes the last two steps of PC synthesis *via* the PE methylation pathway, induces the UPR in both the absence and presence of choline (when choline can be used to synthesize PC *via* the CDP-choline pathway), whereas loss of *CHO2* only upregulates the UPR in media lacking choline (14).

As the committed step of PC-DRP, we hypothesized that *GPC1*, like other genes involved in PC metabolism, might be important for ER bilayer homeostasis, especially given its role in recycling and remodeling PC following lipid turnover events. In the current study, we show that Gpc1 is a target of the UPR upon both proteotoxic and bilayer stress. Further, we report that the loss of *GPC1* upregulates the UPR *via* a mechanism involving bilayer stress. Loss of *GPC1* increases sensitivity to UPR inducers, as expected if *GPC1* is an essential part of the response to these compounds. These results provide evidence for the importance of Gpc1 (and PC-DRP) in the maintenance of optimal ER bilayer composition.

## Results

### Gpc1 is localized to the ER

**Figure 2:**
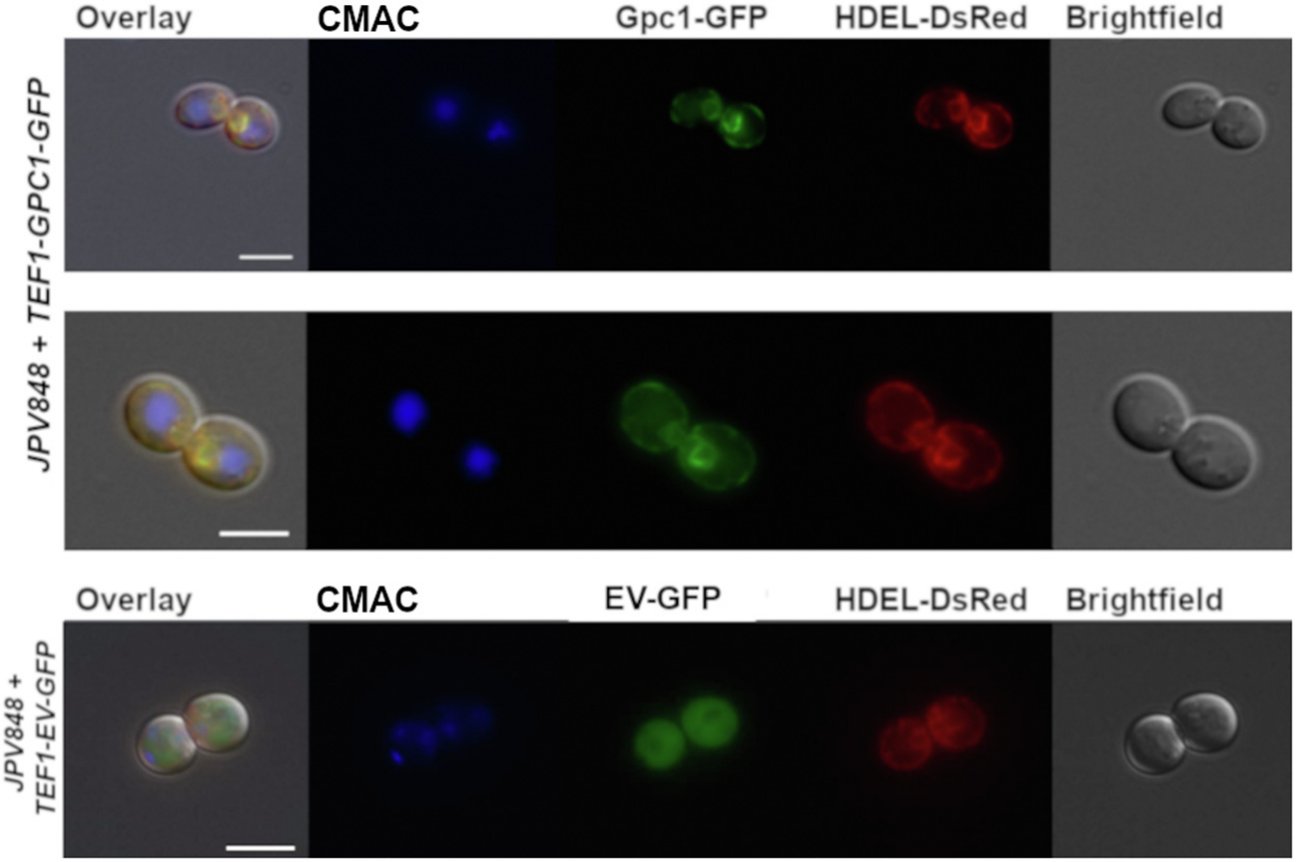
**Gpc1 is localized to the ER.** From *left to right* for *top* two images: Overlay of red and green channels; CMAC; Gpc1 with C-terminal GFP tag; HDEL-DsRed ER marker; Brightfield. Nikon TiE microscope, 100× objective. From *left to right* for bottom image: Overlay of red and *green channels*; CMAC; EV with C-terminal GFP tag; HDEL-DsRed ER marker; Brightfield. Nikon TiE microscope, 100× objective. Bar = 5 μm. Of 1000 cells viewed (JPV848 + *TEF1-GPC1-*GFP), 850 (85%) expressed both Gpc1-GFP and HDEL-DsRed, which colocalize. Representative images are shown.

High-throughput studies have generally suggested an ER and cell periphery localization for Gpc1 (37, 38, 39), but those studies were not optimized for Gpc1 imaging. To reassess Gpc1 localization, we transformed wild-type yeast with the integrative plasmid pAM40-HDEL-DsRed (40) to produce strain JPV882 for ER visualization. Strain JPV882 was then transformed with centromeric plasmid *TEF1*-*GPC1*-GFP for simultaneous Gpc1 visualization. Cells in the log growth phase displayed a typical ER perinuclear and cortical membrane pattern throughout the cell. Roughly 85% of 1000 cells examined exhibited colocalization of Gpc1-GFP with HDEL-DsRed. Representative images are shown in Figure 2. As a control, we show an image of JPV882 transformed with the empty vector containing GFP (EV-GFP), which resulted in a diffuse signal with no distinct ER pattern.

### Loss of Gpc1 blocks incorporation of radiolabeled C^14^-choline-GPC into membrane PC

**Figure 3:**
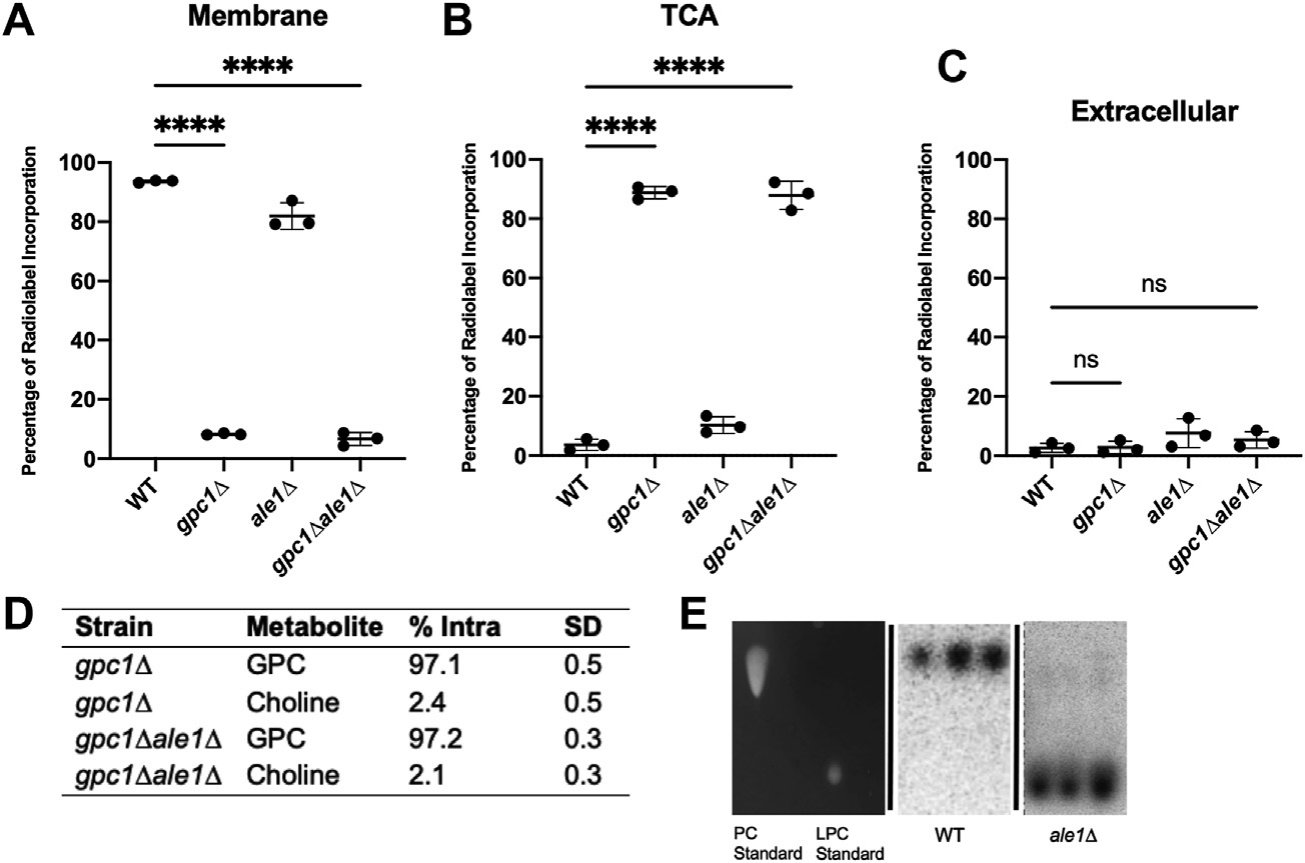
**Loss of Gpc1 blocks incorporation of radiolabeled C**^**14**^**-choline-GPC into membrane PC *via* PC-DRP and results in a buildup of intracellular GPC.***A–C*, cells harboring a plasmid containing the *Candida albicans* Git3 transporter were grown in the presence of C^14^-choline-GPC for 60 min and harvested. Phospholipids were extracted from the membrane (*A*) and water-soluble metabolites extracted from TCA-treated (*B*) and extracellular (*C*) fractions as described in “Experimental procedures.” Each symbol represents a biological replicate. Data are presented as a percentage of total counts incorporated into each fraction. *D*, percent GPC and choline found in intracellular fractions (% Intra). WT and *ale1Δ* are not included in the table as all C^14^-choline-GPC counts were taken up in the membrane fraction for those strains. *E*, TLC plate of WT and *ale1Δ* radiolabeled lipids, as compared to PC and lyso-PC standards. For all statistical comparisons, significance was determined *via* two-tailed *t* test. ∗∗∗∗*p* ≤ 0.0001.

To illustrate the impact of Gpc1 activity on PC synthesis through PC-DRP, we performed radiolabeling (Fig. 3). In an improvement to our previous method that relied on the endogenous Git1 transporter with low affinity for GPC (23), we used a plasmid overexpressing the high-affinity *Candida albicans* GPC transporter Git3 (41) to drive flux of radiolabeled GPC into the cell. Following radiolabeling with C^14^-choline-GPC, cells were treated with TCA *via* our established method (23, 42) and separated into a membrane fraction and water-soluble extracellular and intracellular fractions. For these experiments, *ale1Δ* and *gpc1Δale1Δ* strains were included to confirm the role of the LPA acyltransferase Ale1 in PC-DRP.

After 1 h, the label is almost completely gone from the medium (extracellular fraction) in all strains (Fig. 3). In the WT strain, label was primarily found in the membrane fraction in the form of PC as determined by TLC analysis (Fig. 3*E*), indicative of flux through PC-DRP. In *ale1Δ*, the label was also found primarily in the membrane fraction (Fig. 3*A*), but in the form of lyso-PC (Fig. 3*E*), as expected since PC-DRP is blocked at the lyso-PC acyltransferase step. In *gpc1Δ* and *gpc1Δale1Δ* strains, in contrast, radiolabel primarily accumulated intracellularly in water-soluble compounds (primarily GPC, with lesser amounts of free choline). These data illustrate that in the absence of Gpc1, GPC molecules produced through PC turnover events cannot be reincorporated into membrane PC.

### Both proteotoxic stress by tunicamycin exposure and bilayer stress by inositol limitation induce *GPC1* expression *via* the UPR

**Figure 4:**
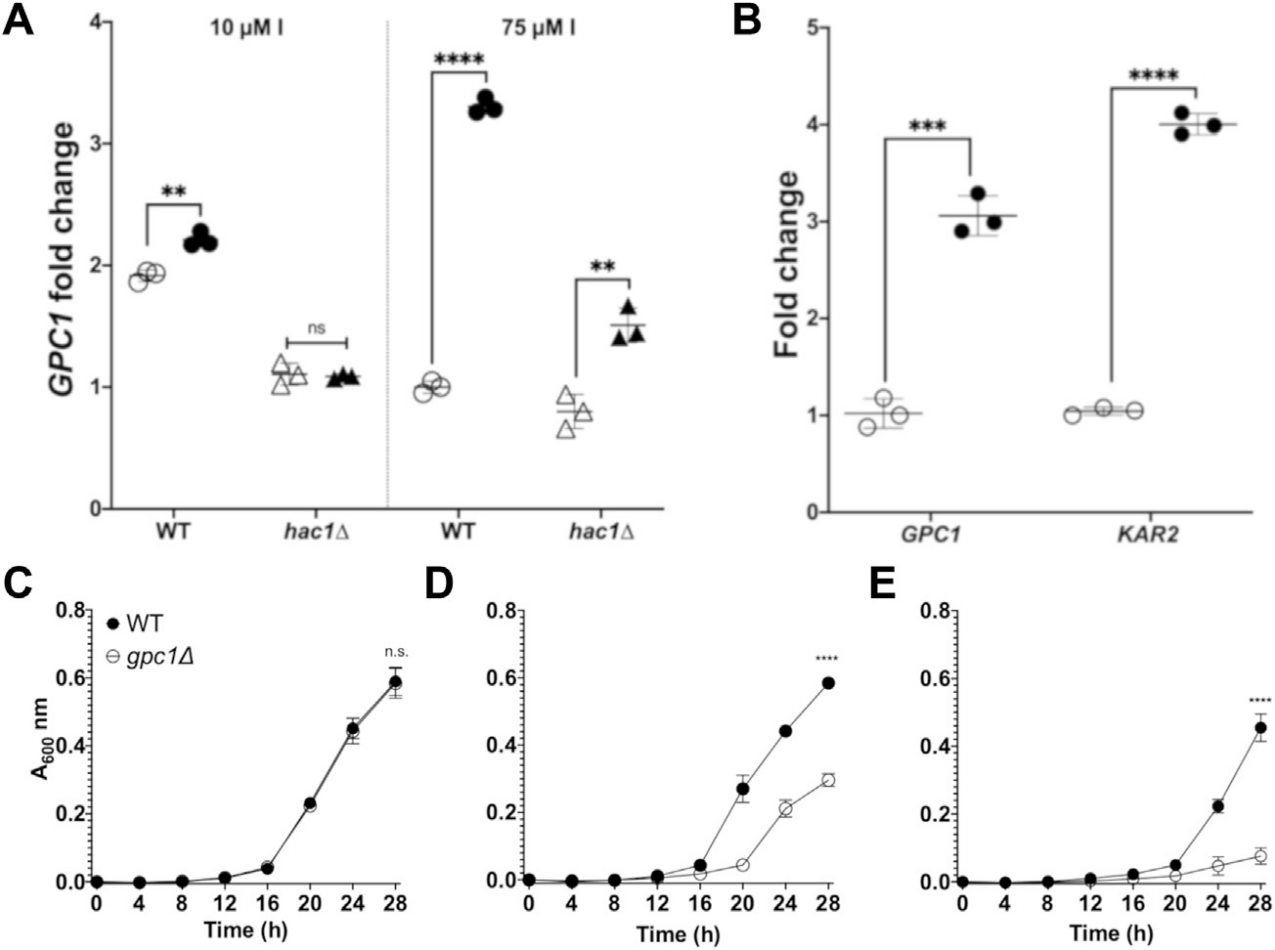
**Both proteotoxic stress by tunicamycin exposure and bilayer stress by inositol limitation induce *GPC1* expression *via* the UPR. A *gpc1Δ* strain is more sensitive to Tm.***A*, the indicated strains were grown in YNB media with low (10 μM) or 75 μM inositol. Cells were grown to log phase and exposed to DMSO (vehicle control) or tunicamycin I (1 mg/L) for 1 h. Closed symbols denote Tm exposure and open symbols denote DMSO-only controls. RNA was extracted and qRT-PCR was performed as described under “Experimental procedures.” The data are normalized to WT in I+ without Tm. Each symbol represents a biological replicate performed in technical triplicate and error bars represent standard deviation. Statistical analyses were conducted using a 2-tailed *t* test. *B*, WT yeast were grown in I+ media. Cells were grown to log phase and then exposed to DMSO or 1 mg/L Tm for 1 h. *Closed circles* denote WT exposed to Tm and open symbols denote DMSO-only WT controls. RNA was extracted and *GPC1* and *KAR2* message was quantified. For each gene, expression is normalized to the condition without Tm. Each symbol represents a biological replicate performed in technical triplicate. *C–E*, strains were grown in YNB media without inositol (I-) at the following concentrations of Tm: 0 mg/L (*C*), 0.25 mg/L (*D*), and 0.5 mg/L (*E*). Growth was determined using a Molecular Devices SpectraMax i3, at 30 °C with intermittent shaking. Data are displayed as the mean and standard deviation of four replicates per strain. Statistical analyses were conducted using a 2-tailed *t* test. ∗∗*p* ≤ 0.01; ∗∗∗*p* ≤ 0.001; ∗∗∗∗*p* ≤ 0.0001.

Tunicamycin is one of three proteotoxic UPR inducers used in these studies, the others being DTT and canavanine (5, 33, 43). We have shown previously that inositol limitation induces the transcription of *GPC1* (23). Inositol limitation is also a condition that induces the UPR *via* bilayer stress (3, 5, 13). In Figure 4*A* we examined the impact of tunicamycin (Tm) treatment on *GPC1* expression under both low (10 μM) and normal (75 μM) inositol conditions. A *hac1Δ* mutant was utilized to determine the dependency of the response (*GPC1* expression) to these conditions on the UPR (see Fig. 1*B*). Because the *hac1Δ* mutant is an inositol auxotroph, the complete absence of inositol was not possible. As shown in the left panel, Tm induces a small but significant increase in *GPC1* expression in low-inositol conditions, where *GPC1* expression is already elevated two-fold when compared to 75 μM inositol. In contrast, when cells are grown in 75 μM inositol, Tm exposure results in a roughly 3.5-fold increase in *GPC1* expression (Fig. 4*A*, right panel). Importantly, under both conditions, a *hac1Δ* mutant displays no change or only a partial change in expression upon Tm exposure, indicating that the UPR is largely responsible for the upregulation. Also apparent from the data is that the increase in *GPC1* expression that occurs upon inositol limitation (compare open circles across panels) is Hac1-dependent (compare open triangles across panels). This indicates that both proteotoxic (Tm exposure) and bilayer stress (inositol limitation) regulate the expression of *GPC1 via* the UPR. A separate experiment (Fig. 4*B*) confirms that the UPR target *KAR2*, which encodes a molecular chaperone (1, 7), is upregulated by Tm in tandem with the upregulation of *GPC1* message.

### A *gpc1Δ* mutant is more sensitive to Tm

Since *GPC1* expression is upregulated upon Tm exposure, we reasoned that the growth of a *gpc1Δ* mutant may be abrogated in the presence of Tm concentrations that are sublethal to the WT strain. As shown in Figure 4, *C*–*E*, a *gpc1Δ* mutant displays growth indistinguishable from WT in the absence of Tm, but slower growth than WT at 0.25 and 0.5 mg/L Tm. Note that these growth experiments were performed in I- media, which induces the UPR *via* bilayer stress and results in altered regulation of many lipid biosynthetic genes in addition to other signaling pathways (3, 5, 13, 18, 44). Under I+ conditions, the sensitivity of the *gpc1Δ* strain to Tm is less apparent (data not shown).

### *GPC1* expression is upregulated by DTT *via* the UPR, and a *gpc1Δ* strain is more sensitive to DTT

**Figure 5:**
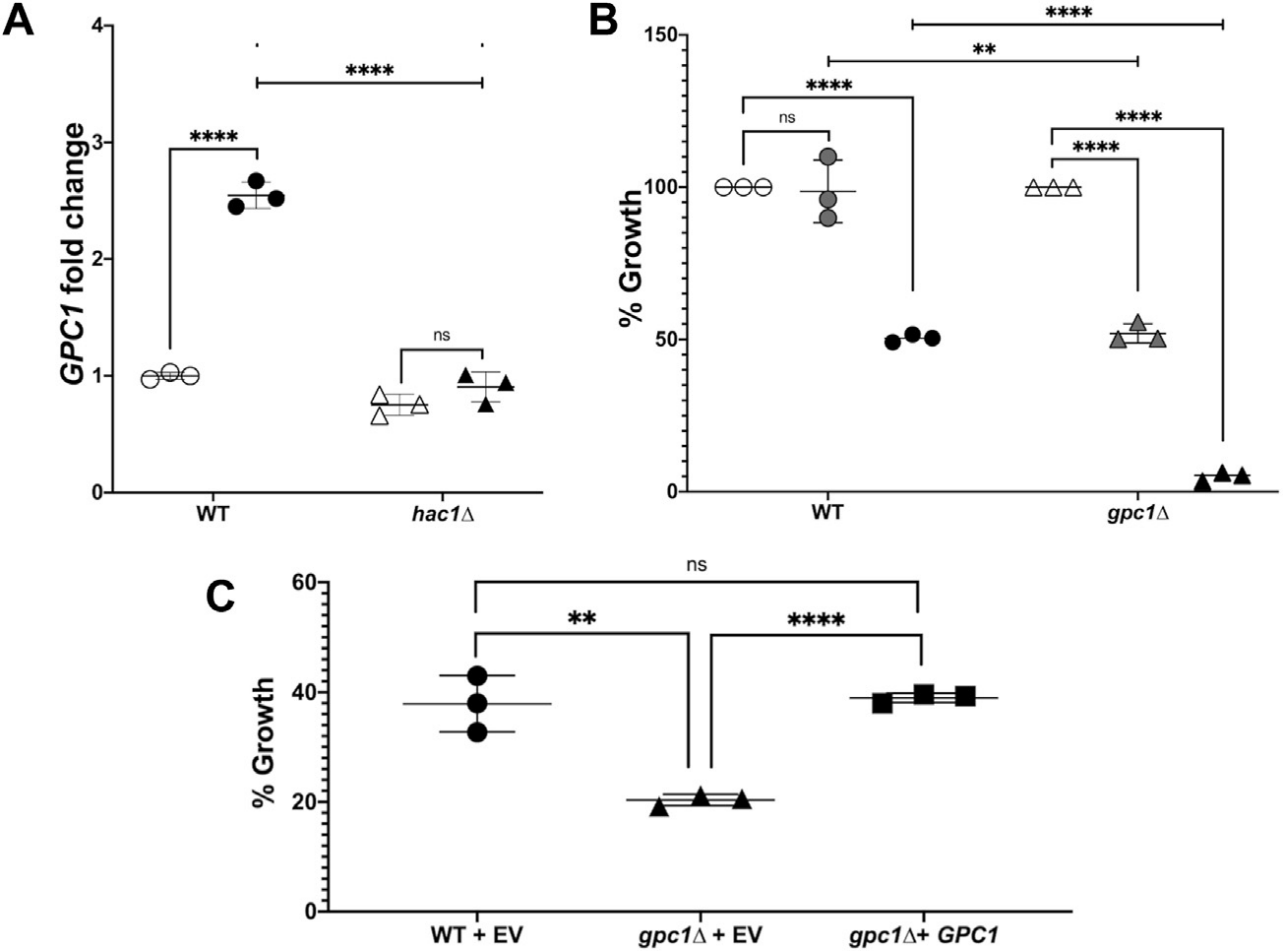
***GPC1* expression is upregulated by DTT *via* the UPR, and a *gpc1Δ* strain is more sensitive to DTT.***A*, strains were grown to log phase in I+ media. Cells were then exposed to 3 mM DTT or an equivalent volume of water for 1 h. Closed symbols denote DTT exposure and open symbols denote controls. RNA was extracted and qRT-PCR was performed as described under “Experimental procedures.” The data are normalized to WT without DTT. Each symbol represents a biological replicate performed in technical triplicate, and error bars represent standard deviation. Statistical analyses were conducted using a two-tailed *t* test. *B*, the indicated strains were grown to log phase in I+ media in biological triplicate. Cultures were exposed to DTT at each of the following concentrations for 1 h: 0 mM (open symbols), 2 mM (*gray symbols*), and 3 mM (*black symbols*). Cultures were pelleted and washed to remove the drug, reinoculated at equivalent densities, and grown for an additional 17 h. Growth is displayed as % growth relative to untreated conditions based on A_600nm_ readings, and error bars represent standard deviation. Statistical analyses were conducted using a 2-tailed *t* test. *C*, complementation assay. The growth assay was performed as described for *B* in I+ media with 0 mM or 3 mM DTT, except cells were grown on I+ media lacking uracil to maintain the empty vector (EV) or the plasmid containing *GPC1* under its native promoter. Error bars represent standard deviation. Statistical analyses were conducted using a 2-tailed *t* test. ∗∗*p* ≤ 0.01; ∗∗∗∗*p* ≤ 0.0001.

We next tested DTT exposure, which reduces disulfide bonds and induces the UPR *via* proteotoxic stress. In this case, cells were only grown in the presence of inositol (I+ media) to eliminate the upregulation of the UPR that occurs upon inositol limitation. As shown in Figure 5*A*, 1 h exposure of WT cells to 3 mM DTT results in a roughly 2.5-fold increase in *GPC1* expression. This upregulation, like that caused by Tm exposure and inositol deprivation (Fig. 4*A*), is Hac1-dependent.

Because DTT is highly toxic to cells, we performed a reinoculation growth experiment following acute exposure to assess DTT sensitivity. WT cells display little or no growth inhibition following 1 h exposure to 2 mM DTT and a roughly 50% inhibition in growth upon exposure to 3 mM DTT (Fig. 5*B*). In contrast, a *gpc1Δ* mutant strain is more sensitive, exhibiting roughly 50% less regrowth upon 2 mM DTT exposure, and complete growth inhibition at 3 mM DTT. A single copy of *GPC1* on a plasmid restores WT growth to a *gpc1Δ* mutant strain, confirming the *GPC1*-dependence of the growth phenotype (Fig. 5*C*).

### *GPC1* expression is upregulated by canavanine exposure *via* the UPR, and a *gpc1Δ* strain is more sensitive to canavanine

**Figure 6:**
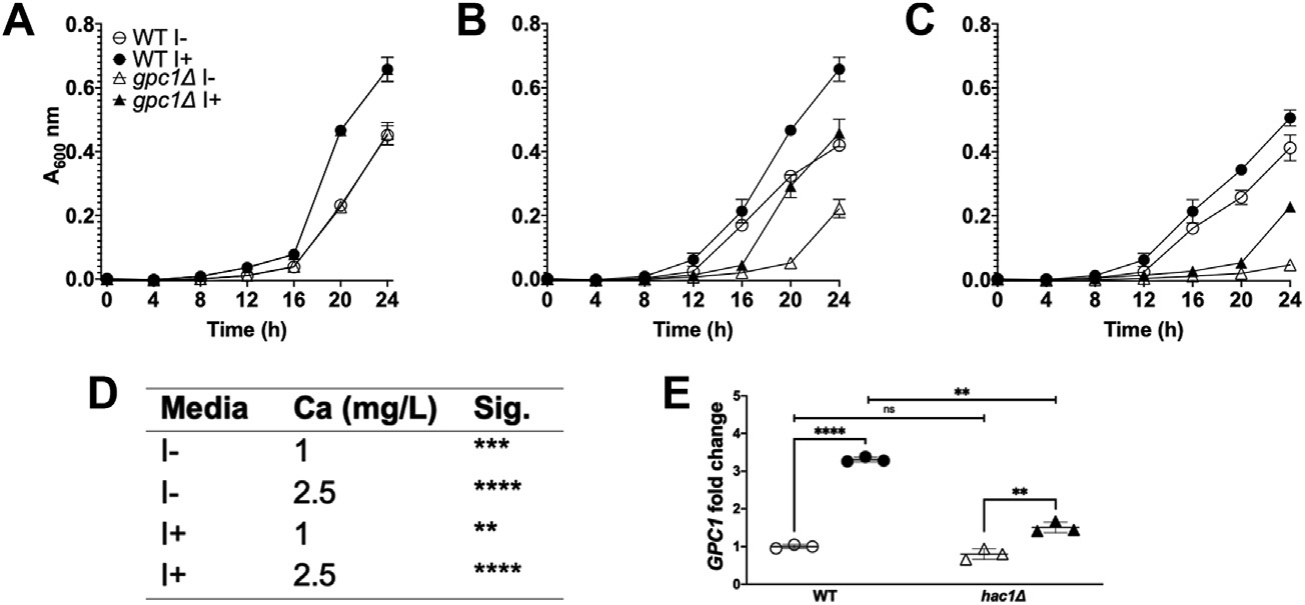
***GPC1* expression is upregulated by canavanine exposure *via* the UPR, and a *gpc1Δ* strain is more sensitive to canavanine.***A*–*C*, the indicated strains were grown in I− or I+ media, without arginine (arg-), on a Molecular Devices SpectraMax i3, at 30 °C with intermittent shaking. The following concentrations of canavanine were used: 0 mg/L (*A*), 1 mg/L (*B*), and 2.5 mg/L (*C*). Data are displayed as the mean and standard deviation of four replicates per strain. Note that in (*A*), WT and *gpc1Δ* for each respective media type are overlapping. *D*, statistical comparisons between strains for the 24 h timepoint are shown. Statistical analyses were conducted using a 2-tailed *t* test. *E*, the indicated strains were grown in I+ media lacking arginine. Cells were grown to log phase and exposed to 2.5 mg/L canavanine or an equivalent volume of water for 1 h. Closed symbols denote cells exposed to canavanine and open symbols denote water-only controls. Each symbol represents a biological replicate performed in technical triplicate, and error bars represent standard deviation. Statistical analyses were conducted using a 2-tailed *t* test. RNA was extracted and qRT-PCR was performed as described under “Experimental procedures.” The data are normalized to WT without canavanine. ∗∗*p* ≤ 0.01; ∗∗∗∗*p* ≤ 0.0001.

Canavanine, a toxic analog of arginine, induces the UPR (5, 45). Like Tm and DTT, exposure of cells to canavanine results in *GPC1* transcriptional upregulation in a Hac1-dependent manner (Fig. 6*E*). Also, similar to what occurs with Tm and DTT, the growth of the *gpc1Δ* mutant is abrogated as compared to WT at both 1 mg/ml and 2.5 mg/ml canavanine (Fig. 6, *A*–*D*). These growth differences were apparent in cells grown both in the presence (I+) and absence (I-) of inositol.

### Loss of Gpc1 induces the UPR

**Figure 7:**
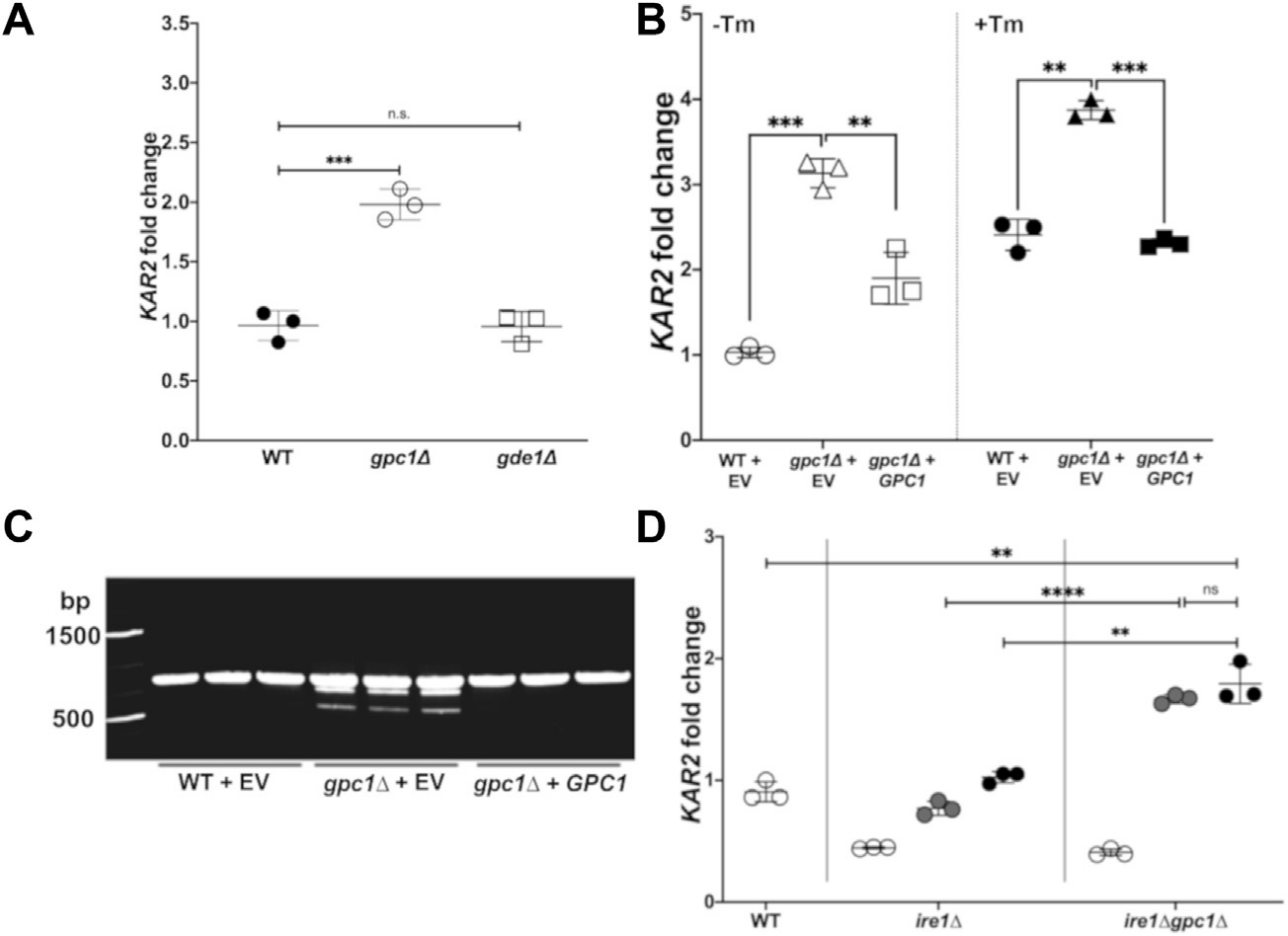
**Disruption of PC synthesis *via* PC-DRP by deletion of Gpc1 induces the UPR *via* bilayer stress**. *A*, the indicated strains were grown to log phase in I+ media. RNA was extracted and qRT-PCR was performed as described under “Experimental procedures.” The data are normalized to WT. Each symbol represents a biological replicate performed in technical triplicate, and error bars represent standard deviation. *B*, the indicated strains were grown to log phase in I+, ura- YNB media, with either 0 mg/L Tm or 1 mg/L Tm exposure for 1 h. DMSO was used as vehicle control for the 0 mg/L Tm condition. Closed symbols denote Tm exposure and open symbols denote DMSO-only controls. RNA was extracted and qRT-PCR was performed as described under “Experimental procedures.” The data are normalized to WT + EV in 0 mg/L Tm. Each symbol represents a biological replicate performed in technical triplicate, and error bars represent standard deviation. *C*, RNA was extracted and cDNA was converted as described under “Experimental procedures.” Generic PCR was set up using primers from Di Santo *et al*. (51). PCR products were visualized *via* agarose gel electrophoresis. *D*, the indicated strains were grown to log phase in I+, his- YNB media. Strains contained one of the following plasmids: empty vector (EV) (*open circles*), Ire1 lacking the luminal domain (Ire1ΔLD) (*gray circles*), or full-length Ire1 (Ire1) (*black circles*) (5). RNA was extracted and qRT-PCR was performed as described under “Experimental procedures.” The data are normalized to WT containing EV. ∗∗*p* ≤ 0.01; ∗∗∗*p* ≤ 0.001; ∗∗∗∗*p* ≤ 0.0001.

We have shown that *GPC1* is a transcriptional target of the UPR in cells undergoing proteotoxic stress by Tm, DTT, or canavanine (Figs. 2, 4 and 5) and bilayer stress induced by inositol limitation (Fig. 4*A*). We next asked the converse question: Does loss of the ER-localized acyltransferase Gpc1, the committed step in the conversion of GPC to PC, cause induction of the UPR? Expression of *KAR2*, a molecular chaperone (46, 47, 48, 49, 50), and splicing of the transcription factor *HAC1* (51, 52, 53, 54) are commonly used methods for monitoring UPR induction. As shown in Figure 7*A*, a *gpc1Δ* mutant exhibits an increase in *KAR2* message as compared to WT. The *GPC1* dependence of the induction is addressed in Figure 7*B*, where a plasmid-borne copy of *GPC1* complements the increase in *KAR2* associated with the loss of *GPC1*, restoring *KAR2* expression to near-wild type levels (left panel). The impact of the simultaneous assaults of Tm exposure and loss of Gpc1 on UPR induction was also examined. In the presence of Tm (Fig. 7*B*, right panel), where *KAR2* expression is already upregulated (compare open *versus* closed circles across panels), the absence of Gpc1 results in a further uptick in *KAR2* expression. This additive impact on UPR induction is complemented by a plasmid-borne copy of *GPC1.*

A *gpc1Δ* strain also displays increased *HAC1* splicing (Fig. 7*C*), a second method for monitoring UPR induction (51). The increase in *HAC1* splicing, visualized by the appearance of lower molecular weight splicing products, is complemented by a plasmid-borne copy of *GPC1* (Fig. 7*C*).

### Induction of the UPR in *gpc1Δ* is not due to GPC buildup

All of our data to this point, especially that obtained using the Ire1ΔLD construct (Fig. 7*D*), are consistent with the hypothesis that blocking the conversion of GPC to PC through PC-DRP in *gpc1Δ* is the reason for this induction of the UPR. However, another technical possibility is that the buildup of GPC in the cell, as is experienced by the *gpc1Δ* mutant (Fig. 3), is the cause. To assess this, we employed a *gde1Δ* strain, which lacks the other metabolic output for GPC, namely breakdown into choline and glycerol-3-phosphate (G-3-P) (Fig. 1*A*) (20, 21). As shown in Figure 7*A*, a *gde1Δ* mutant does not exhibit an increase in *KAR2* expression. This result is consistent with our other data supporting the interpretation that induction of the UPR in *gpc1Δ* is the result of blocking PC synthesis through PC-DRP.

### Loss of Gpc1 induces the UPR in an Ire1LD-independent manner, indicating bilayer stress

Given that loss of Gpc1 induces the UPR (Fig. 7, *A*–*C*), we next performed experiments to determine the mechanism of induction. An Ire1ΔLD construct, which contains only the first 12 residues of the normally 495-residue Ire1 (Fig. 1*B*) luminal domain (LD), is unresponsive to proteotoxic stress but remains responsive to bilayer stress (5). Single-copy plasmids containing either full-length Ire1 or Ire1ΔLD (5, 55) were transformed into a *ire1Δgpc1Δ* strain (Fig. 7*D*). As expected, similar upregulation of *KAR2* occurred when *ire1Δgpc1Δ* contained either full-length Ire1 or Ire1ΔLD (right panel). These results indicate that loss of Gpc1 induces the UPR *via* bilayer stress. A comparison between the middle and right panels serves as a control for this system and confirms that loss of Gpc1 induces the UPR. Note that the empty vector controls (no Ire1 present) represent the lack of a functional UPR.

### Loss of Gpc1 impacts the lipidome under bilayer stress

Thus far, we have provided data in support of two related yet converse findings linking Gpc1 to the UPR. *GPC1* expression is upregulated by the UPR in response to both proteotoxic and bilayer stress (Figs. 4*A*, 5*A* and 6*E*). Conversely, loss of Gpc1 induces the UPR *via* a mechanism involving bilayer stress, as it does not require the luminal domain of Ire1 (Fig. 7*D*). We next performed lipidomic experiments to examine the impact of PC-DRP on PC species and glycerophospholipid content under stressed and unstressed conditions as a function of Gpc1 dosage.

**Figure 8:**
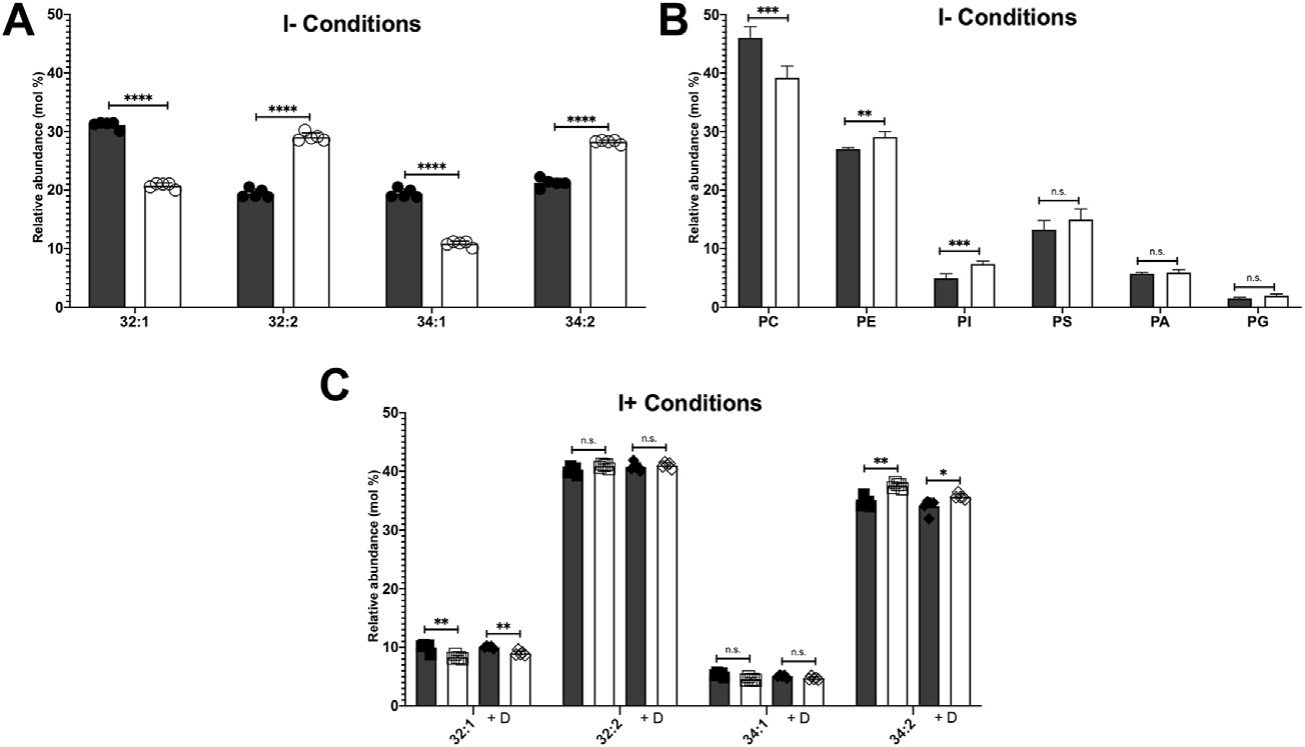
**Loss of Gpc1 alters the PC species profile upon bilayer stress.** Cells were grown to log phase and 20 ODU were harvested. DTT (1 h, 2 mM) was used for treatments in I+ media where indicated. Lipids were extracted as described under “Experimental procedures” and used for ESI-MS^2^ analysis. Data are displayed as relative abundance (molecular percentages). *Gray bars* indicate WT and white bars indicate *gpc1Δ*. Two-tailed T-tests were used to determine significance. Five biological replicates were used per strain as represented by each symbol. Error bars represent standard deviation. *A*, relative abundance of the four primary PC species in I- media. *B*, relative abundance of major glycerophospholipids in I- media. *C*, relative abundance of the four primary PC species in I+ media with DTT treatment where indicated. ∗*p* ≤ 0.05; ∗∗*p* ≤ 0.01; ∗∗∗*p* ≤ 0.001; ∗∗∗∗*p* ≤ 0.0001.

Consistent with our previous findings, under the bilayer stress of inositol limitation a *gpc1Δ* strain exhibited a decrease in monounsaturated PC species (32:1 and 34:1) and an increase in di-unsaturated PC species (32:2 and 34:2) that is absent in the presence of inositol (Fig. 8, *A* and *C*). An examination of relative glycerophospholipid composition indicated a slight decrease in total PC content in the *gpc1Δ* strain and slight compensatory increases in PE, PI, and PS (Fig. 8*B*). Together, these findings indicate a connection between *HAC1*-dependent upregulation of *GPC1* and lipidomic changes in PC species that are dependent upon Gpc1.

We also examined the impact of proteotoxic stress imposed by DTT on PC species, as DTT caused an increase in *GPC1* expression (Fig. 5*A*). In this case, we detected little or no change in PC species either in the presence or in the absence of DTT or the presence or absence of Gpc1 (Fig. 8*C*). This result was not unexpected, as others have similarly been unable to detect whole-cell lipidomic changes in response to proteotoxic stressors such as DTT in cells grown in synthetic media, despite the upregulation of several lipid biosynthetic genes (56). One interpretation of this result is that any lipidomic changes caused by 1-h DTT treatment are too transient to be detected. In contrast, the bilayer stress of inositol limitation occurs throughout growth. Further, inositol limitation results in transcriptional changes in multiple lipid biosynthetic genes *via* the Henry regulatory circuit (57, 58), in addition to expression changes in a subset of lipid-related genes, like *GPC1*, under the control of the UPR. In that metabolic context, Gpc1-dependent lipidomic changes may simply be easier to detect.

Overall, the lipidomic findings indicate a connection between the upregulation of *GPC1 via* the UPR *via* bilayer stress, and lipidomic changes in PC species that are dependent upon Gpc1. We further interpret these results to suggest that these alterations in PC species are likely the cause of the bilayer stress engendered by the loss of Gpc1 (Fig. 7).

### Growth analysis indicates a negative genetic interaction between *GPC1* and *IRE1*

**Figure 9:**
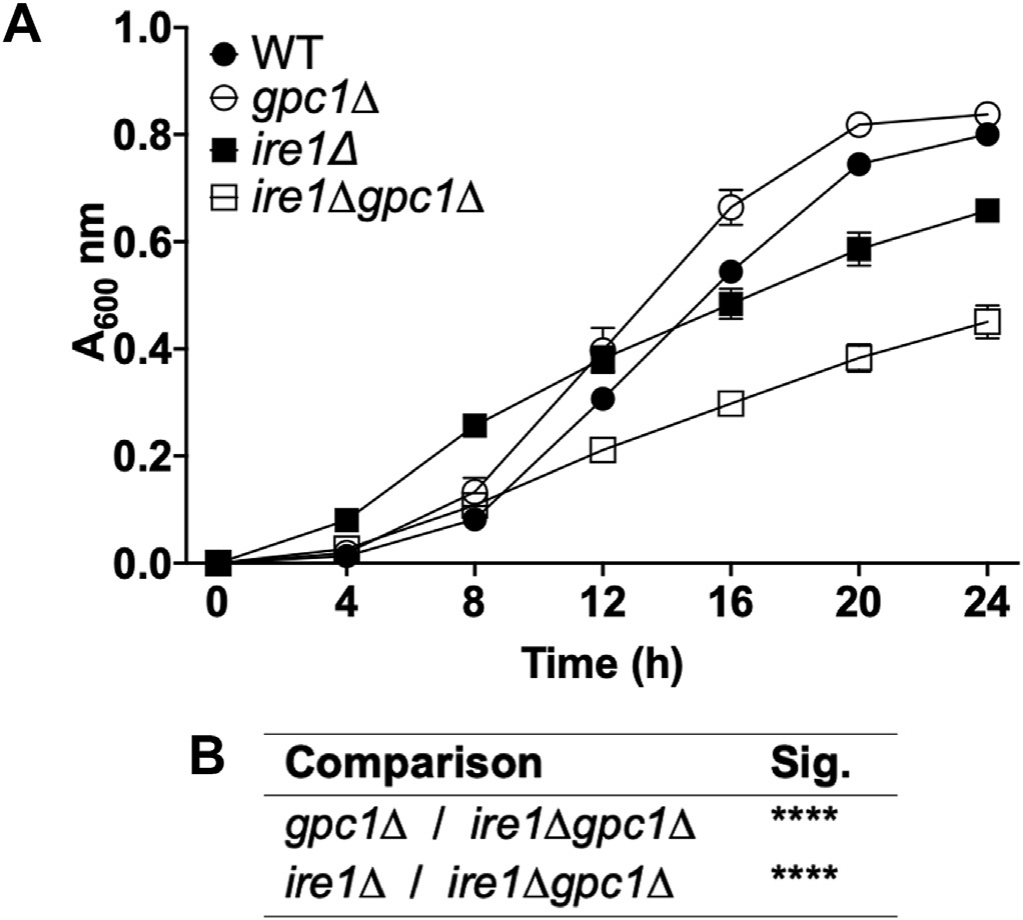
**An *ire1Δgpc1Δ* double mutant exhibits a negative genetic interaction.***A*, the indicated strains were grown in media containing 75 μM inositol on a Molecular Devices SpectraMax i3, 30 °C with intermittent shaking. Data are displayed as the mean and standard deviation of eight replicates per strain. *B*, statistical comparisons between strains for the 24 h time point are shown. Statistical analyses were conducted using a 2-tailed *t* test. ∗∗∗∗*p* ≤ 0.0001.

In synthetic media, *gpc1Δ* exhibits similar growth to WT (Fig. 9). The *ire1Δ* mutant displays a slight growth defect as would be expected from a strain lacking a major stress response gene and as reported by others (59, 60). However, *ire1Δgpc1Δ* demonstrates a greater growth defect than either individual knockout, indicating a negative genetic interaction between this UPR transducer and *GPC1*. This provides further evidence that Gpc1 is involved in a shared biological process with Ire1 and that their loss results in a synergistic impairment (61).

## Discussion

The maintenance of membrane homeostasis requires the coordinated control of multiple metabolic activities including biosynthetic enzymes involved in lipid synthesis, phospholipases involved in lipid turnover, and acyltransferases involved in lipid remodeling. The metabolism of the major glycerophospholipid PC is, likewise, complex. In addition to the two major pathways of PC biosynthesis, the CDP-choline and the PE methylation pathways, PC is subject to deacylation and remodeling *via* PC-DRP (Fig. 1*A*). Other metabolic pathways also impinge on PC biosynthesis. Besides being used for PC-DRP, the GPC produced through PC deacylation can be degraded by the glycerophosphodiesterase Gde1 to produce glycerol-3-p and choline (21, 62). Additionally, phosphatidic acid and choline are produced *via* Pld1/Spo14 (63, 64) hydrolysis of PC. The free choline produced through both of these catabolic processes can be recycled into PC synthesis *via* the CDP-choline pathway. In this complex metabolic context, we report that Gpc1, catalyzing the first acylation step of PC-DRP, plays a significant role in ER bilayer homeostasis. *GPC1* is both a transcriptional target of the UPR in response to proteotoxic and bilayer stress (Figs. 4*A*, 5*A* and 6*E*), and its loss induces the UPR *via* bilayer stress (Fig. 7). Further, the bilayer stress is attributable to detectable lipidomic changes in PC species (Fig. 8). The confirmation that Gpc1 is ER-localized is consistent with its role in ER membrane homeostasis (Fig. 2).

Bilayer stress and unfolded protein stress are detected by Ire1, the key transducer of the UPR in yeast. The luminal domain (LD) of Ire1 is key to the detection of unfolded proteins (proteotoxic stress) (5, 65). The transmembrane helix in combination with an amphipathic helix immediately adjacent to the transmembrane helix of Ire1 is thought responsible for detecting lipid bilayer stress. An early report identified 19 lipid-metabolism-related UPR targets (66), and subsequent studies added to that number (5, 15, 67). Fewer genes have been identified as being both targets of the UPR and to induce the UPR upon their loss (14, 15, 68). Focusing on PC metabolism, *OPI3* and *CHO2* (grown in the absence of exogenous choline) fall into this category (14, 67). The data presented here add *GPC1* to that list.

Bilayer stress can be induced by a variety of signals, including a degree of membrane lipid saturation, sterol content, the protein-to-lipid ratio, and inositol depletion (yeast) (69). Separate from the UPR^LBS^ response, inositol limitation also controls the expression of a host of phospholipid biosynthetic genes *via* the Henry regulatory circuit (58, 70) involving the Ino2/Ino4 transcriptional activators and the Opi1 transcriptional repressor. Importantly, the upregulation of *GPC1* that occurs upon inositol limitation (23) (Fig. 4*A*) occurs through the UPR (Hac1) and not the Henry regulatory circuit (Fig. 4). These findings are consistent with the fact that the promoter region of *GPC1* contains three unfolded protein response elements (UPREs), which correspond to Hac1 binding sites as identified through YeasTract (71). The UPREs are located at −305, −182, and −666 bp upstream of the start codon of *GPC1*.

We used an improved method to follow flux through PC-DRP *in vivo*. This method employed constitutive heterologous expression of CaGit3, which encodes a high-affinity transporter for GPC. In previous studies (23) we relied upon the endogenous ScGit1 transporter which has a low affinity for GPC (preferring GPI) and required growth under low phosphate conditions to induce expression and a long-term labeling strategy (72, 73). The results of Figure 3 show clearly that flux through PC-DRP requires Gpc1.

PC-DRP provides the cell with a means for PC resynthesis and the potential for PC remodeling. We have repeated previous findings and report that loss of Gpc1 leads to significant changes to the PC species profile under the bilayer stress of inositol limitation, namely a decrease (roughly 20%-30%) in monounsaturated PC species (32:1PC and 34:1PC) and a concomitant increase in di-unsaturated PC species (32:2PC and 34:2PC). We also find a slight decrease in relative PC content in the absence of PC-DRP. We argue that these lipidomic changes are responsible for the bilayer stress observed in a *gpc1Δ* strain (Fig. 7). The new data reported here showing that *GPC1* expression is largely Hac1-dependent allows us to link these lipidomic changes to the UPR.

We also probed the possibility that we could detect Gpc1-dependent lipidomic changes in response to proteotoxic stress but were unable to do so (Fig. 8*C*). We were not surprised by these results due to a recent comprehensive analysis (56). The authors reported only minor changes to the *S. cerevisiae* glycerophospholipid lipidome, including little or no change to PC, upon short-term DTT or Tm exposure for cells grown in synthetic media (the growth and proteotoxic stress conditions used in this study). To explain this finding in light of the studies (5, 55) reporting changes in lipid-related gene expression under the same conditions, we can speculate that small, localized, dynamic changes originating in the ER lipidome are not readily detected by whole cell analysis. Subcellular fractionation could theoretically increase the sensitivity of the lipidomic analysis upon ER stress, but it is currently technically challenging to rapidly isolate clean ER membranes *via* a method that does not, in itself alter the lipidome, especially given the proliferation of membrane contact sites (74, 75) to this organelle. Current protocols to isolate clean ER require multiple steps and several hours (76).

Nonetheless, phenotypic evidence that UPR upregulation of *GPC1* by proteotoxic stress has functional consequences is provided by the finding that a *gpc1Δ* mutant displays increased sensitivity to DTT, canavanine, and Tm (Figs. 3, 4 and 6). Furthermore, our growth studies demonstrating a negative genetic interaction between *GPC1* and *IRE1* link the genes to a common process (Fig. 9).

Gpc1, a 52 kDa protein with eight predicted transmembrane domains (77), bears no sequence similarity to known acyltransferases or transacylases and has been designated a new protein family (UniProtKB - P48236) (78). Gpc1 sequence homologs are lacking in vertebrates but are found in other organisms, including plants (22), and medically important pathogenic fungi such as *C. albicans* (41). Gpc1-like activity has also been detected in *Xanthomonas campestris* (79) and the Mitis group Streptococci—which includes major human pathogens (80). Additionally, a recent study has placed Gpc1 in a large superfamily that includes a distant member (less than 10% sequence identity) to TMEM 164, an arachidonate-preferring lyso-plasmalogen acyltransferase (81). Future studies will be aimed at identifying key residues involved in Gpc1 function.

## Experimental procedures

### Strains and media

*S. cerevisiae* strains (Table 1) were grown aerobically at 30 °C in a roller drum or a culture shaker. A Thermo Scientific BioMate160 spectrophotometer was used to assess growth *via* A_600nm_ measurements. Yeast peptone dextrose (YPD) media was used for the maintenance of strains. Yeast nitrogen base (YNB) media with 2% glucose was prepared as described (82), with inositol content varied as indicated. YNB dropout media was used to select yeast strains transformed with plasmids. Plasmids were maintained as *Escherichia coli* freezer stocks and transformation was performed as described (23). See Table 1 for a list of plasmids and strains used in this study.

**Table 1:** Strains and plasmids used in this work

Strain or plasmid		Genotype/Description	Source or Ref.
Strains			
JPV #			
848	WT BY4742	MATα his3Δ1 leu2Δ0 lys2Δ0 ura3Δ0	(86)
846	WT BY4742 *GPC1*-3xHA	BY4742, *GPC1*-3xHA	(23)
865	*gpc1Δ*	BY4742, *gpc1*::*KanMX*	(86)
671	*hac1Δ*	BY4742, *hac1*::*KanMX*	(86)
878	*ire1Δ*	BY4742, *ire1*::*KanMX*	(86)
877	*ire1Δgpc1Δ*	BY4742, *ire1*::*KanMX gpc1::LEU2*	This study
887	*hac1Δgpc1*	BY4742, *hac1*::*KanMXgpc1::LEU2*	This study
601	*ale1Δ*	BY4742, *ale1*::*KanMX*	(86)
888	*gde1Δ*	BY4742, *gde1*::*KanMX*	(86)
832	*gpc1Δale1Δ*	BY4741, *ale1::LEU2 gpc1::KanMX*	(22)
882	WT pAM40-HDEL-DsRed	MATα containing integrative pAM40-HDEL-DsRed plasmid (JVE426)	This study; (40)
Plasmids			
JVE #			
145	pRS316	Single-copy plasmid with *URA3*	
415	pRS316-*GPC1*	*GPC1* and native promoter (750 bp upstream) inserted into pRS316	This study
421	pRS313	Single-copy plasmid with *HIS3*	
422	pGT330	*IRE1* inserted into pRS313	(5)
423	pGT201	*IRE1* with luminal domain deletion (Ire1ΔLD) inserted into pRS313	(5)
418	p416-*GIT3*	*GIT3* inserted into p416, under the control of *TEF1* promoter	This study
426	pAM40-HDEL-DsRed	Plasmid with *URA3* marker and pRS306 backbone containing HDEL sequence as ER localization control	(40)
393	p416	Multi-copy plasmid with *URA3* marker and *TEF1* promoter	
392	*TEF1*-*GPC1*-GFP	Plasmid with *LEU2* marker, containing *GPC1* under *TEF1* promoter	This study

### Plasmid construction

*GPC1* and 750 bp upstream were amplified from the genome and cloned into a single-copy pRS316 vector using XmaI and NotI cut sites to generate pRS316-*GPC1* (Table 1). Ampicillin was used to select for retention of the plasmid in *E. coli*. Uracil dropout plates were used to select for retention of the plasmid in *S. cerevisiae*.

### Growth analyses

Overnight cultures were used to inoculate a 96-well plate at A_600nm_ = ∼0.01. Plates were incubated at 30 °C, with intermittent shaking prior to each reading using a Molecular Devices SpectraMax i3 instrument. Hourly A_600nm_ readings were taken and time zero values were subtracted from each timepoint to reflect overall growth. Each curve reflects a minimum of four biological replicates. Where indicated, media contained tunicamycin (Sigma Aldrich), DTT (Fisher BioReagents), or canavanine (Sigma Aldrich).

To perform reinoculation growth assays, 5 ml cultures in YNB media containing 75 μM inositol were grown in triplicate to log phase. Next, the indicated experimental drug was added. Following 1 h of drug exposure, cells were harvested, washed, and used to restart new overnight cultures. Following overnight (∼17 h) growth at 30 °C in a drum roller, A_600nm_ readings were obtained using a Thermo Scientific BioMate160 spectrophotometer. Reinoculation growth is presented as a percentage of growth in drug-treated cultures *versus* untreated cultures. T-tests were performed to determine significance, with the following notation used: ∗*p* ≤ 0.05; ∗∗*p* ≤ 0.01; ∗∗∗*p* ≤ 0.001; ∗∗∗∗*p* ≤ 0.0001.

### Microscopy

Wild-type yeast (JPV848) were transformed with pAM40-HDEL-DsRed, *TEF1*-*GPC1*-GFP, and/or *TEF1*-EV-GFP plasmids (Table 1) (40). pAM40-HDEL-DsRed was integrated into WT (JPV848) to produce strain JPV882 (Table 1). *TEF1*-*GPC1*-GFP was maintained in WT *via* selective media that allowed for the expression of *GPC1* under a constitutive promoter. Cells were grown to early log phase, spiked with 25% YPD, and grown to mid-log phase. Cells were washed, then spotted onto agarose-coated glass slides. Cells were imaged using a Nikon TiE inverted microscope (Nikon Instruments, Tokyo, Japan), an Orca Flash 4.0 cMOS camera (Hammamatsu) and 100× objective (NA 1.45). Image acquisition was obtained using NIS-Elements software (Nikon).

### Radiolabeling with C^14^-choline-GPC

Strains containing p416-*GIT3*, the plasmid harboring the *C. albicans* GPC transporter encoded by *GIT3* (41), were grown to log phase in YNB media lacking uracil. Cultures were provided with C^14^-choline-GPC (≅200,000 cpm/ml) (American Radiolabeled Chemicals 3880) and allowed to grow for 1 h. Cultures were then harvested, and the cell pellets were treated with 5% TCA for 20 min on ice. The suspension was pelleted and aliquots of both the pellet (containing lipids) and the water-soluble TCA extract were subjected to liquid scintillation counting as described (23, 42). The identity of the labeled lipid in the pellet was confirmed as PC by TLC analysis (42). The TLC plate shown in Figure 3*E* was imaged using a Typhoon 8200 phosphorimager.

### RNA extraction and qRT-PCR

RNA was extracted from one ODU of cells using the hot phenol extraction protocol (83). RNA integrity was confirmed on an agarose gel. A Thermo Scientific NanoDrop One was used to quantify RNA concentrations. Total RNA (1 μg) was converted to cDNA using a Thermo Scientific Verso cDNA synthesis kit. cDNA was converted at 42 °C for 1 h followed by a 2-min reverse transcriptase step at 65 °C. cDNA conversion was confirmed *via* generic PCR set up with *SNR17* primers, followed by visualization on an agarose gel. qRT-PCR was performed with a Thermo Scientific Maxima SYBR Green/ROX qPCR Master Mix (2×) using primers listed in Table 2.

**Table 2:** qRT-PCR and splicing assay primers

Gene name	Primer	Sequence (5′-3′)
*SNR17*	Forward	TTG ACT CTT CAA AAG AGC CAC TGA
	Reverse	CGG TTT CTC ACT CTG GGG TAC
*GPC1*	Forward	TGT GTG GCA TAT GGA TTC GT
	Reverse	GTA ATC CTT ATC ATT CAC GG
*OLE1*	Forward	GGC TAG AGC TGA TAT TAC CG
	Reverse	GCG TTT CTG TAA TCA GTT GG
*KAR2*	Forward (47)	AAG ACA AGC CAC CAA GGA TG
	Reverse (47)	AGT GGC TTG GAC TTC GAA AA
*HAC1*	Forward (51)	ACG ACG CTT TTG TTG CTT CT
	Reverse (51)	TCT TCG GTT GAA GTA GCA CAC

All data were normalized to *SNR17* using a ΔΔCT analysis method. Unless otherwise noted, qRT-PCR data are graphed as averages of three technical replicates for each of the three independent cultures ± SD. Two-sided T-tests assuming unequal variance were performed to determine significance. The following notation is used for all figures: ∗*p* ≤ 0.1; ∗∗*p* ≤ 0.01; ∗∗∗*p* ≤ 0.001; ∗∗∗*p* ≤ 0.0001.

### Lipidomics analysis

Cultures were grown to the logarithmic phase in YNB media and 20 ODUs were harvested. For lipid extractions, the cell pellets were treated with 5% TCA for 20 min on ice. Following centrifugation, the supernatant was discarded, and cell pellets were incubated at 60^°^C for 60 min with 1 ml of ESOAK (95% ethanol, diethyl ether, H_2_O, pyridine, NH_4_OH [28–30%]; 15:5:15:1:0.036 v/v/v/v/v) (84). The tubes were centrifuged to pellet the debris, and 1 ml of lipid-containing supernatant was transferred to fresh tubes containing 2.5 ml of chloroform/methanol (2:1) and 0.25 ml of 0.1 M HCl. Following vortexing and low-speed centrifugation, the bottom layers containing glycerophospholipids were dried under N_2_ (23). Glycerophospholipids were then analyzed using ESI-MS^2^ at the Kansas Lipidomics Research Center as described previously (85). The internal standards were 0.6 nmol di12: 0-phosphatidylcholine (PC), 0.6 nmol di24: 1-PC, 0.6 nmol 13: 0-lysoPC, 0.6 nmol 19: 0-lysoPC, 0.3 nmol di12: 0-phosphatidylethanolamine (PE), 0.3 nmol di23:0-PE, 0.3 nmol 14: 0-lysoPE, 0.3 nmol 18: 0-lysoPE, 0.3 nmol di14: 0-phosphatidic acid (PA), 0.3 nmol di20: 0(phytanoyl)-PA, 0.3 nmol di14: 0-phosphatidylglycerol (PG), 0.3 nmol di20: 0(phytanoyl)-PG, 0.2 nmol di14: 0-phosphatidylserine (PS), 0.2 nmol di20: 0(phytanoyl)-PS, 0.23 nmol 16: 0 to 18: 0-phosphatidylinositol (PI) and 0.16 nmol di18: 0-PI. The signals from these standards were quantified and used in normalization to account for ionization differences among classes.

### HAC1 splicing assay

RNA was extracted and total cDNA was generated from log phase cultures as described in “RNA extraction and qRT-PCR.” For analysis of *HAC1* splicing, the following intron-flanking primers were used for generic PCR: forward, ACGACGCTTTTGTTGCTTCT; reverse, TCTTCGGTTGAAGTAGCACAC (51). *HAC1* PCR products were then assessed *via* agarose gel electrophoresis to determine whether they appeared at the projected unspliced (819 bp) or spliced (567 bp) band size (51).

### Statistical analysis

Two-tailed *t* test analyses were performed to establish significance using GraphPad Prism 8.

## Data availability

Data available upon request to Jana Patton-Vogt (pattonvogt@duq.edu)

## Conflict of interest

The authors declare that they have no conflicts of interest with the contents of this article. The content is solely the responsibility of the authors and does not necessarily represent the official views of the National Institutes of Health.
